# Delayed Surgery and Adenosine, Lidocaine, and Mg^2+^ Immunomodulatory Therapy Improve Joint Recovery in a Sex-Specific Manner After Anterior Cruciate Ligament Reconstruction in a Rat Model

**DOI:** 10.1177/03635465251383556

**Published:** 2025-10-23

**Authors:** Jodie L. Morris, Hayley L. Letson, Peter C. McEwen, Geoffrey P. Dobson

**Affiliations:** † Heart, Sepsis and Trauma Research Laboratory, College of Medicine and Dentistry, James Cook University, Townsville, Australia; ‡ Orthopaedic Research Institute of Queensland, Townsville, Australia; § College of Medicine and Dentistry, James Cook University, Townsville, Australia; Investigation performed at the Heart, Sepsis and Trauma Research Laboratory, College of Medicine and Dentistry, James Cook University, Townsville, Australia

**Keywords:** anterior cruciate ligament (ACL) reconstruction, surgical timing, graft healing, sex differences

## Abstract

**Background:**

The timing for anterior cruciate ligament (ACL) reconstruction (ACLR) and strategies to enhance postoperative healing remain controversial, with little known regarding potential sex-specific differences. Perioperative adenosine, lidocaine, and Mg^2+^ (ALM) therapy has been shown to promote joint tissue healing in an experimental model of early ACLR; however, an early-late comparison of ALM’s effects on postoperative recovery remains to be investigated.

**Purpose:**

To examine the effect of delayed surgery in males and females, and the effect of ALM therapy to augment joint tissue healing.

**Study Design:**

Controlled laboratory study.

**Methods:**

After noninvasive ACL rupture, adult male (n = 38) and female (n = 39) Sprague-Dawley rats were randomly divided into early (3-day delay) or delayed (14-day delay) ACLR surgery groups, and to receive ALM therapy or saline. During ACLR, an ALM or saline intravenous 1-hour infusion was commenced before first incision, and an intra-articular bolus of ALM or saline was administered at surgery end. Animals were monitored to 28 days postoperatively, and pain, functional recovery, inflammation and joint tissue repair markers, and histopathology were assessed.

**Results:**

In the first postoperative week, delayed ACLR was associated with a lymphocyte-driven immune response, with less systemic inflammation compared with early ACLR. This response was associated with faster recovery of body weight, and less joint pain and swelling in both sexes. At 28 days postoperatively, graft and adjacent joint tissue healing appeared more advanced in females than males, regardless of surgical timing. In both sexes, ALM blunted synovial levels of inflammatory mediators (tumor necrosis factor–α and interleukin-1β), reduced injury markers in articular cartilage, and improved graft healing, evidenced from increased expression of tissue repair markers and bony ingrowth at the graft-tunnel interface.

**Conclusion:**

Delayed versus early ACLR improves joint recovery, and ALM further augments healing by blunting the early immunoinflammatory response in both sexes, irrespective of surgical timing.

**Clinical Relevance:**

Delaying ACLR has the advantage of reducing the pre- and postoperative joint inflammatory environment, with improved outcomes. Perioperative ALM therapy may augment joint healing in both sexes after ACLR.

Anterior cruciate ligament (ACL) reconstruction (ACLR) surgery remains the gold-standard treatment for active individuals after ACL tear or rupture, with females being more prone to injury than males.^43,59^ Despite major advances in surgical and physical therapy methods, 2% to 35% of patients develop postoperative arthrofibrosis,^6,24,37,57^ up to 20% of patients will experience a reinjury,^45,59^ 65% do not return to their preinjury active lifestyle,^2,18^ and up to 90% of individuals will develop posttraumatic osteoarthritis (PTOA) within 15 years.^5,15,61^

The timing of ACLR surgery is considered a key factor in determining patient outcomes, although the optimal timing remains controversial.^43,53^ The early versus delayed ACLR debate has persisted for >4 decades,^∥^ with the continued lack of consensus perhaps reflecting the growing appreciation of the complexity and multifactorial aspects underlying ACL graft failure and long-term postoperative complications. While there is general agreement that ACLR should be scheduled for a minimum of 3 weeks after injury to allow inflammation and swelling to subside,^53^ for many patients constraints within both the private and public health care systems necessitate delays of months to years for surgical intervention.^47,63^ Recent studies have suggested that to achieve successful long-term clinical and functional outcomes, individualized approaches to surgical management should be guided by the inflammatory state of the ACL-ruptured knee, rather than applying a one-size-fits-all recommendation for injury-to-surgery time.^11,16,25,53^ However, very little is known about the spatial and temporal differences of the immunoinflammatory response and joint tissue healing after ACL injury and ACLR surgery, and even less regarding potential sex differences in these processes.^39,42,44^

Adenosine, lidocaine, and Mg^2+^ (ALM) therapy is a novel systems-acting drug that has been shown to blunt systemic and local inflammation, reduce immune dysfunction, and improve healing in experimental models of total knee arthroplasty,^40,41^ skeletal muscle trauma,^23^ ischemic stroke,^62^ abdominal surgery,^7^ hemorrhagic shock,^13,14,19,31,32,34,58^ traumatic brain injury,^33^ thermal burns,^8^ and sepsis.^20,21^ Previous studies have shown that ALM promotes joint tissue healing in both sexes after a 3-day surgical delay in a preclinical model of early ACLR.^42,44^ However, comparison of ALM’s effects on postoperative recovery after delayed ACLR has not been characterized. Using a clinically relevant rat model of ACL rupture and reconstruction, we aimed to (1) compare the effect of early (3-day delay) and delayed (14-day delay) ACLR surgery on local and systemic immunoinflammatory responses and joint healing processes in males and females up to 28 days postoperatively, and (2) examine the effect of ALM perioperative therapy to accelerate recovery and joint tissue repair after delayed ACLR. We hypothesized that (1) compared with early surgery, delaying ACLR would reduce the magnitude of the postoperative systemic inflammatory response in both sexes, and (2) ALM therapy would augment joint tissue repair by modulating early immunoinflammatory processes, regardless of sex and timing.

## Methods

### Experimental Design

**Figure 1.**
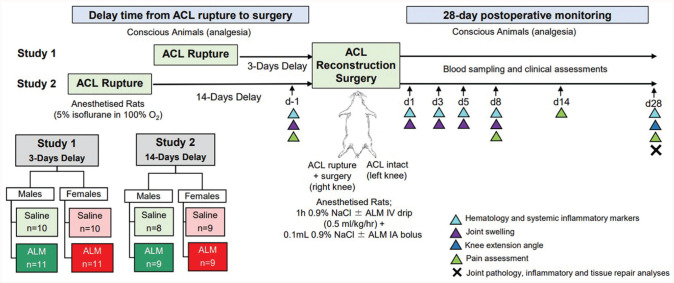
Study protocol schematic. ACL, anterior cruciate ligament; ALM, adenosine, lidocaine, and Mg^2+^; d, day; IA, intra-articular; IV, intravenous.

A schematic of the experimental design is shown in Figure 1. To compare the effect of surgical timing on postoperative recovery after early (study 1) and delayed (study 2) ACLR, we undertook novel comparative analysis of previously published study 1 inflammatory and tissue repair data.^42,44^ Given the differences in the metabolic rates of rats and humans,^48^ we chose 3 and 14 days’ delay after surgery to reflect early and delayed ACLR, which approximates 2 and 12 months in humans. Study protocols were reviewed and approved by the Institutional Animal Ethics Committee (No. A2684) and the US Animal Care and Use Review Office and are reported according to the Animal Research: Reporting of In Vivo Experiments guidelines.

Conventional 16-week-old male (n = 38; 441 ± 38 g) and female (n = 39; 246 ± 19 g) Sprague-Dawley rats were randomly divided into early (3 days post-ACL rupture) or delayed (14 days post-ACL rupture) ACLR surgery groups, and to receive ALM therapy (males: 3-day delay [n = 11], 14-day delay [n = 9]; females: 3-day delay [n = 11], 14-day delay [n = 9]) or saline treatment (controls) (males: 3-day delay [n = 10], 14-day delay [n = 8]; females: 3-day delay [n = 10], 14-day delay [n = 9]). Animals were acclimated for a minimum of 7 days before experimentation; were housed in individually ventilated cages (Tecniplast) with access to environmental enrichment, standard rodent pellets (Specialty Feeds), and water ad libitum; and were maintained in a 14- to 10-hour dark-light cycle under controlled temperature (21°C-22°C) and humidity (65%-75%).

Noninvasive ACL rupture of the right hindlimb was performed on anesthetized animals using a custom apparatus, as described previously.^39^ At 3 (study 1) or 14 (study 2) days after rupture, animals underwent remnant-sparing ACLR surgery on the right knee using a tail tendon autograft,^42,44^ with postoperative assessment over 28 days. All surgeries were performed by a single surgeon (P.C.M.) with blinding of the surgical team and data analyst (J.L.M.) to treatment assignment. Immediately before recovery from anesthesia, animals received Carprieve (5 mg/kg, subcutaneous) for pain relief, with analgesic administered 24 hours thereafter, according to pain scores of individual animals. Animal numbers for each metric are shown in Appendix Table A1 (see the Appendix available in the online version of this article).

### Treatment

The ALM treatment group received a 0.5-mL/kg/h intravenous (IV) infusion for 1 hour via the left femoral vein, commencing immediately before skin incision (adenosine: 18.7 mM; lidocaine: 34.6 mM; MgSO_4_: 41.5 mM in 0.9% NaCl).^44^ Immediately after capsule closure and before skin closure, animals also received an intra-articular (IA) bolus of ALM (0.1 mL; adenosine: 1 mM; lidocaine: 3 mM; MgSO_4_: 2.5 mM in 0.9% NaCl).^44^ Saline control animals received a 0.9% NaCl IV drip with an IA bolus of 0.9% NaCl.

### Clinical Recovery

Time to recover preoperative (immediately before ACLR) body weight and time to bear full weight were assessed. In addition, joint pain was assessed using mechanical paw withdrawal thresholds in the operated and nonoperated knees before surgery (day 1) and at days 1, 5, 8, 14, and 28 postoperatively, as previously described.^39^ Joint swelling was assessed 1 day before surgery (day 1) and 1, 3, 5, and 8 days after ACLR surgery, using methods described previously.^39^ In addition, knee extension angles^39^ and temporal-spatial gait variables were measured 28 days postoperatively, using methods described previously.^42^

### Hematology and Inflammatory Mediator Assessments

In addition to sampling from the temporary venous catheter on the day of surgery (1 hour postoperatively), blood (0.5 mL) was collected from the tail vein of anesthetized animals at day 1 and at 1, 3, 5, and 28 days after ACLR surgery. A complete blood cell count was performed (VetScan HM5 analyzer; Abaxis), and inflammatory cytokines and chemokines were measured in plasma and joint synovial wash (interleukin [IL]-6, IL-1β, IL-4, IL-18, tumor necrosis factor [TNF]-α, interferon-inducible protein [IP]–10, monocyte chemoattractant protein [MCP]–1, macrophage inflammatory protein 1 alpha [MIP-1α], and vascular endothelial growth factor) using custom Milliplex Rat Cytokine/Chemokine Magnetic Bead Panels (Abacus ALS), as described previously.^39^ Baseline ranges for hematological parameters (10 per sex) and inflammatory mediators (8 per sex) were determined in healthy male and female animals.

### Flow Cytometry

Leukocyte phenotyping was performed using cell surface and intracellular markers (Appendix Table A2) and flow cytometry, as previously described.^44^ Absolute numbers of circulating B cells, T-cell subsets (T_Helper_, T_Cytotoxic_, and T_Regulatory_ cells), natural killer (NK) cells, neutrophils, and classic and nonclassic monocytes were measured in peripheral blood before surgery (day 1), on the day of surgery (1 hour postoperatively), and at 1, 3, and 5 days postoperatively.^44^ In addition, leukocyte subsets were assessed in draining lymph nodes (popliteal and inguinal^22,64^) of operated knees at day 28 postoperatively and compared with baseline levels in draining lymph nodes of healthy, age-matched male and female animals. Absolute cell counts were determined from the inclusion of CountBright Plus Absolute Counting beads (Invitrogen). Data were analyzed with FlowJo analysis software Version 10 (FlowJo LLC, Inc).

### Histology

At 28 days postoperatively, animals were euthanized to assess joint pathology and tissue repair markers. Macroscopic scoring of the intact joint, the articular surfaces, and capsular and synovial tissue was performed using a modified grading system, as described previously.^41^ Synovial lavage was performed on operated and nonoperated knees.^39^ Quadriceps atrophy was assessed by comparing wet weights of quadriceps muscles from operated and nonoperated hindlimbs relative to total body weight. ACL graft and articular cartilage samples were collected for gene expression analysis. The remaining joint tissue was fixed in 4% paraformaldehyde for 48 hours, decalcified with 14% ethylenediaminetetraacetic acid, processed, paraffin embedded, and sectioned (4 µm) in the coronal plane across the entire expanse of the joint, with sections approximately 80 µm apart.

Semiquantitative evaluation of joint histopathology was performed by a blinded investigator (J.L.M.) on a minimum of 3 sections per knee, with section scores combined and averaged for each knee. Hematoxylin and eosin (H&E) staining was used to assess synovitis, fibrotic changes and cell infiltration within the medial joint capsule surrounding the site of incision for ACLR, using previously described scoring criteria.^41^ To assess ACL graft healing, the graft midsubstance was scored across 5 variables, and graft-to-bone healing within the femoral and tibial bone tunnels across 4 variables (Table A3, available online), with each criterion scored on H&E-, safranin O/fast green (SafO)–, and picrosirius red–stained sections using a 0 (normal tissue architecture) to 3 (severe pathology) scale scoring system. Cartilage degradation within the medial femoral condyle and medial tibial plateau was assessed in SafO-stained sections according to the Osteoarthritis Research Society International (OARSI) scoring scale,^17^ and as described previously.^39^ A score of 10 represented the maximal degree of severity of knee osteoarthritis. Immunohistochemical localization of markers for mesenchymal cells/fibroblasts (vimentin; D21H3; 1:120 dilution), bone remodeling (transforming growth factor beta 1 [TGF-β1]]; EPR21143; 1:120 dilution), myofibroblasts (α–smooth muscle actin [α-SMA]; 1A4; 1:1000), pan-macrophages (CD68; ED1; 1:125 dilution), and myeloid-derived suppressor cells (MDSCs)/M2 macrophages (arginase I; AB_2792410; 1:150 dilution) was performed using methods described previously.^42^

### RNA Isolation and Quantitative Real-time Polymerase Chain Reaction

Total RNA was isolated from ACL graft tissue and articular cartilage and cDNA was prepared by reverse transcription.^39,41^ Real-time polymerase chain reaction with custom-designed primers was used to assess the gene expression of key markers of inflammation (nuclear factor kappa B subunit 1), extracellular matrix (ECM) constituents (collagen type 1 alpha 1 chain [Col1a1], collagen type 2 alpha 1 chain [Col2a1], collagen type 3 alpha 1 chain [Col3a1], fibronectin 1 [Fn1], elastin [Eln], aggrecan [Acan], and cellular communication network factor 2 [Ccn2]), ECM remodeling enzymes (matrix metalloproteinase 9 and 13 [Mmp13] and tissue inhibitor of matrix metalloproteinase 1 [Timp1]), and stem cell/fibroblast activation and differentiation (transforming growth factor beta 1 [Tgfb1], fibroblast growth factor 1 [Fgf1], and actin alpha 2 [Acta2]) (Table A4, available online). Hypoxanthine-guanine phosphoribosyl transferase (Hprt1) was used as the reference gene for normalization. The data show relative expression in operated compared with nonoperated knees of each animal.

### Statistical Analysis

Sample sizes were determined from a priori power analysis using G*Power 3 software (Heinrich Heine University, Düsseldorf) to determine sample size with effect size for outcome measure Nfkb expression in knee joint tissue in a rat model of knee implant surgery (allocation ratio N2/N1 = 1; Cohen *d* = 2.28; = alpha error probability = 0.05; sample size = 5; power [1 − beta error probability] = 0.88). Statistical analyses were performed using GraphPad Prism software (Version 10.3.1). Normality assumptions and equality of variances were assessed using Shapiro-Wilk and Levene tests, respectively. Two-way analysis of variance with the Tukey honestly significant difference test was used for between- and repeated-measures testing within groups. Between-group differences for gene expression and histological assessments were assessed using the Kruskal-Wallis test. The results are expressed as mean ± standard error of the mean unless otherwise stated, with significance set at a *P* value <.05.

## Results

### Preoperative Systemic Inflammation

**Figure 2.**
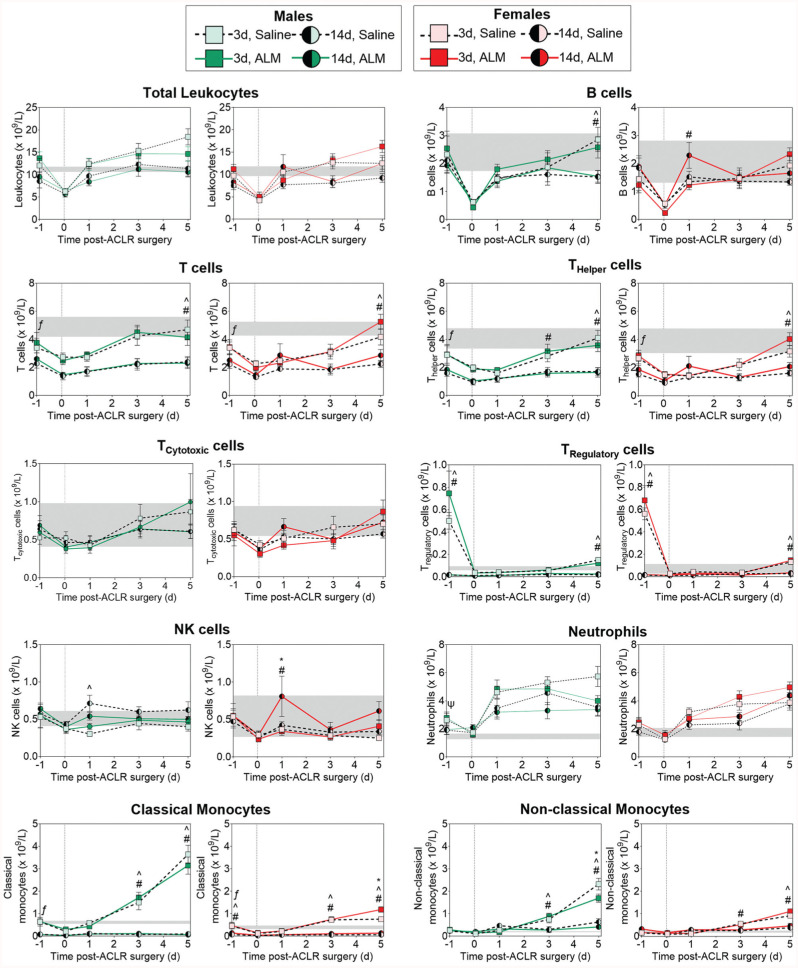
Changes in peripheral blood leukocyte subsets in male and female adenosine, lidocaine, and Mg^2+^ (ALM)–treated and saline control animals after early (3 days) and delayed (14 days) anterior cruciate ligament reconstruction (ACLR) surgery. Data are shown as mean ± SEM. n = 8 to 11 per sex per time point. *P < .05, 3-day alm compared with 3-day saline. ^*P* < .05, 3-day saline compared with 14-day saline. ^#^*P* < .05, 3-day ALM compared with 14-day ALM. ^Ψ^*P* < .05, 3-day saline compared with baseline. ^ƒ^*P* < .05, 14-day saline compared with baseline. Two-way analysis of variance, Tukey post hoc test. The gray shaded areas show the mean ± SEM for healthy baseline animals (8 per sex). The dotted vertical line represents commencement of ACLR surgery (time 0). NK, natural killer.

To compare the systemic inflammatory state immediately prior to ACL surgery (day 1), hematology and plasma inflammatory biomarkers were measured at day 2 (study 1) and day 13 (study 2) after ACL rupture. At 2 days after rupture (early ACLR groups), circulating neutrophils (1.9-fold; *P* = .024) and plasma MCP-1 concentrations (2-fold; *P* = .015) remained elevated above baseline levels in males, while systemic markers of the acute inflammatory response were largely subsided in females (Figure 2; Appendix Figure A1, available in the online version of this article). At 13 days after rupture (delayed ACLR groups), circulating T-cell and classic monocyte numbers were significantly reduced compared with baseline levels in both sexes, and are consistent with a switch from acute to reparative inflammation within the injured joint. Together, these data show timing-specific differences in inflammatory and immune profiles after ACL rupture in males and females.

### Injury Profiles, Operative Metrics, and Clinical Recovery

**Figure 3.**
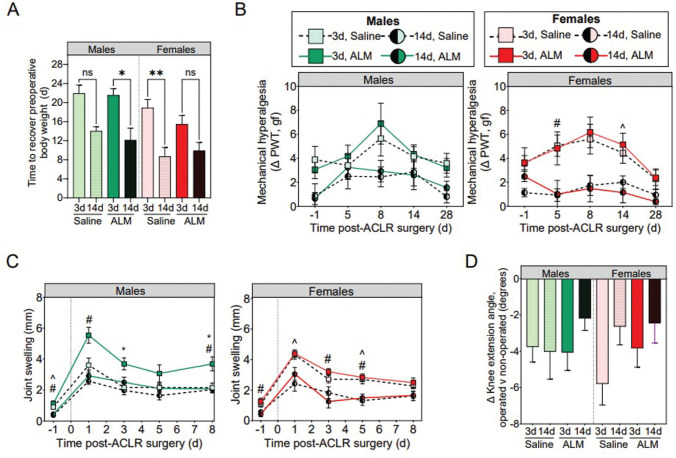
(A) Time to recover preoperative body weight. (B) Mechanical hyperalgesia as an indicator of pain. (C) Joint swelling in the first postoperative week. (D) Knee extension angles at 28 days postoperatively in male and female adenosine, lidocaine, and Mg^2+^ (ALM)–treated and saline control animals after early (3 days) and delayed (14 days) anterior cruciate ligament reconstruction (ACLR) surgery. Data are shown as mean ± SEM. n = 8 to 11 per sex, per time point. (A) *P < .05. **P < .01. Kruskal-Wallis test and Dunn post hoc analysis. (B) and (C) *P < .05, 3-day ALM compared with 3-day saline. ^*P* < .05, 3-day saline compared with 14-day saline. ^#^*P* < .05, 3-day ALM compared with 14-day ALM. Two-way analysis of variance, Tukey post hoc test. The dotted vertical line represents the commencement of ACLR surgery (time 0). ns, not significant; PWT, paw withdrawal threshold.

ACL injury profiles, total surgery times, and intraoperative blood loss were comparable regardless of sex, surgical timing, or treatment groups (Appendix Table A5). No animals showed signs of infection or lameness after ACL rupture or reconstruction surgery. All animals were partial weightbearing unaided immediately after recovery from anesthesia, with natural return to full weightbearing within 2 weeks postoperatively and comparable between sex, surgery, and treatment groups. Compared with early ACLR, body weight was recovered sooner, and joint pain and swelling reduced for both sexes after delayed surgery, with no treatment effect observed for either sex (Figure 3, A-C). Although not statistically significant (*P* = .222), there was a 2.2-fold improvement in knee extension angles in females after delayed versus early surgery, with no significant timing or treatment difference in males (Figure 3D). Compared with early ACLR, step and stride lengths were reduced in males (*P* < .001 and *P* = .005, respectively) and females (*P* = .022 and *P* = .051, respectively) 28 days after delayed surgery (Appendix Table A6, available online). The remaining gait parameters were comparable, regardless of surgical timing. Together, these data suggest that delayed surgery may be associated with slight improvements in clinical and functional recovery in both sexes.

### Postoperative Systemic Inflammatory Responses

To evaluate the effect of timing on sex-specific, surgery-induced systemic inflammatory responses, we compared peripheral blood leukocyte subsets and plasma cytokine and chemokine concentrations after early and delayed ACLR in male and female animals over the first 5 postoperative days (Figure 2; Appendix Figure A1, available online). At 1 hour postoperatively, circulating B cells, T cells, NK cells, neutrophils, and classic monocytes decreased significantly in the peripheral blood of males and females after early and delayed ACLR (Figure 2), which is consistent with the surgery-induced stress response involving rapid recruitment of innate inflammatory cells to the surgical site.^10^ Despite early similarities, distinct sex and surgical timing differences in systemic inflammatory responses were evident from day 1 postoperatively and persisted to day 5. Compared with early ACLR, the magnitude of surgery-induced neutrophil and monocyte mobilization was lower in both males (*P* = .052 and *P* < .001, respectively) and females (*P* = .042 and *P* < .001, respectively) after delayed ACLR (Figure 2), and corresponded to a reduction in plasma concentrations of the inflammatory mediators, IL-1β (*P* = .017) and MCP-1 (*P* = .028), in males (Appendix Figure A1, available online). In addition, circulating classic monocytes and T cells, predominantly T_Helper_ and T_Regulatory_, remained significantly lower than baseline levels to day 5 after delayed ACLR in both sexes (Figure 2), indicating mononuclear cell– rather than inflammatory cell–dominant trafficking to the operated knee. In males, ALM treatment led to a transient spike in plasma IL-6 (3.1-fold; *P* < .0001) and reductions in MCP-1 (1.7-fold; *P* = .015) and IL-4 (3.9-fold; *P* > .05) at 1 hour postoperatively, and decreased circulating NK cells at 24 hours after delayed ACLR, suggesting bolstering of the acute phase response to surgery and dampening of early monocyte and NK cell mobilization. In females, ALM blunted plasma IP-10 levels at 1 hour (1.7-fold; *P* = .004), suggesting decreased recruitment of monocytes and activated lymphocytes, and boosted circulating NK cell (1.9-fold; *P* = .02) numbers at 24 hours postoperatively (Figure 2; Appendix Figure A1, available online). By 28 days postoperatively, peripheral blood leukocyte subset frequencies had returned to baseline levels in both sexes after delayed ACLR. Compared with early ACLR, plasma IL-6 (*P* = .003) and IL-4 (*P* = .001) concentrations were elevated in males, and IL-6 in females (*P* = .014), 28 days after delayed surgery, with ALM boosting IL-6 levels further above baseline levels in females (4.6-fold; *P* = .049) (Appendix Figure A2, available online). Together, these data show sex disparity in the systemic inflammatory response and redistribution of circulating leukocyte subsets after early and delayed ACLR. In addition, ALM appears to dampen surgery-induced systemic inflammation and alter early immune cell mobilization and recruitment in a sex-specific manner.

### Joint Pathology and Inflammatory Profile

**Figure 4.**
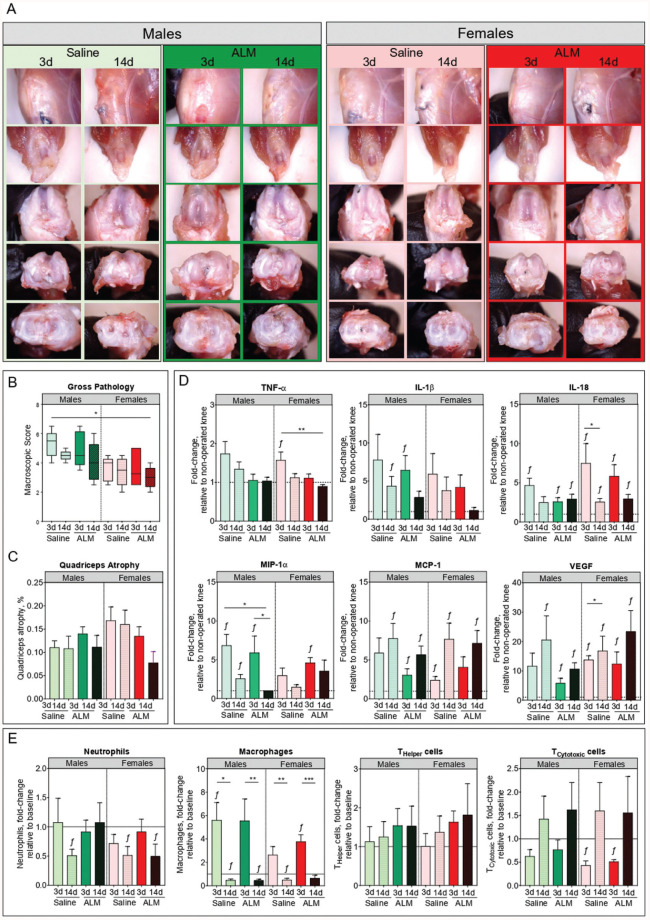
Joint pathology, inflammation, and immune activation in male and female adenosine, lidocaine, and Mg^2+^ (ALM)–treated and saline control animals, 28 days after early (3 days) and delayed (14 days) anterior cruciate ligament reconstruction surgery. (A) Representative images, (B) macroscopic scores, and (C) quadriceps atrophy of dissected operated knees at day 28 postoperatively. (D) Synovial fluid concentrations of inflammatory (tumor necrosis factor alpha [TNF-α], interleukin [IL]–1β, and monocyte chemoattractant protein [MCP]–1), immunoregulatory (macrophage inflammatory protein [MIP]–1α and IL-18), and angiogenic (vascular endothelial growth factor [VEGF]) mediators. (E) Changes in leukocyte subsets within draining lymph nodes (inguinal and popliteal) of operated knees, relative to nonoperated knees. Data are shown as median and IRQ (macroscopic pathology scores) or mean ± SEM. n = 8 to 11 per sex, per time point. *P < .05, **P < .01, and ^ƒ^*P* < .05 compared with nonoperated, anterior cruciate ligament–intact knees. Kruskal-Wallis test, macroscopic scores and quadriceps atrophy. Two-way analysis of variance, Tukey post hoc test, synovial mediators and lymph node immunophenotyping.

Next, we compared joint pathology and inflammatory profiles of operated knees of both sexes, 28 days after early and delayed ACLR surgery. Mild to moderate effusion, joint capsule hypertrophy, synovialization of the graft, and remodeling of articular surfaces were evident in all operated knees, regardless of surgical timing and treatment (Figure 4, A and B). Mild synovial hyperplasia and subsynovial ECM deposition and hypercellularity were evident in the medial capsule of operated knees, with histopathology scores comparable between sex, surgery, and treatment groups (Appendix Figure A3, available online). Quadriceps atrophy, a common response after ACLR surgery, was evident, but was comparable between sex, surgery, and treatment groups (Figure 4C).

Persistence of postoperative joint inflammation and immune reactivity was assessed by levels of inflammatory mediators in synovial fluid and by immunophenotyping of draining lymph nodes of the operated knees at 28 days postoperatively. Compared with early ACLR, synovial TNF-α, IL-1β, IL-18, and MIP-1α levels were reduced in operated knees of both males (1.3-fold, 1.8-fold, 1.9-fold, and 2.7-fold, respectively) and females (1.4-fold, 1.6-fold, 2.9-fold, and 2-fold, respectively) after delayed surgery, although these differences were not statistically significant (Figure 4D). In ALM-treated males, synovial levels of TNF-α (1.3-fold; *P* > .05), IL-1β (1.5-fold; *P* > .05), and MIP-1α (2.6-fold; *P* > .05) were further reduced, and comparable to concentrations in the nonoperated knee after delayed ACLR (Figure 4D). ALM treatment was also associated with a reduction in synovial TNF-α (1.3-fold; *P* > .05) and IL-1β (3.2-fold; *P* > .05) to baseline levels after delayed ACLR in females, as well as increased MIP-1α (2.4-fold; *P* > .05), a chemokine central to tissue repair and remodeling (Figure 4D). Sex, surgical timing, and treatment differences were also apparent in leukocyte subset frequencies of draining lymph nodes of the operated knee, 28 days after ACLR. Compared with intact knees, lymphocyte numbers (predominantly B, activated T_Helper_, T_Cytotoxic_, and NK cells) were reduced, and macrophages were increased in draining lymph nodes after early ACLR, consistent with ongoing immune reactivity in the operated knee (Figure 4E; Appendix Figure A3, available online). In contrast, significantly fewer neutrophils and macrophages were present in the draining lymph nodes of males (*P* = .003 and *P* = .02, respectively) and females (*P* = .012 and *P* = .005, respectively) after delayed surgery, with ALM restoring neutrophil numbers to levels comparable to the intact knee in males (Figure 4E). Together, these data suggest that postoperative joint inflammation resolves sooner in both sexes after delayed compared with early ACLR, and that ALM therapy may enhance these repair processes in a sex-specific manner.

### ACL Graft Healing Profiles

**Figure 5.**
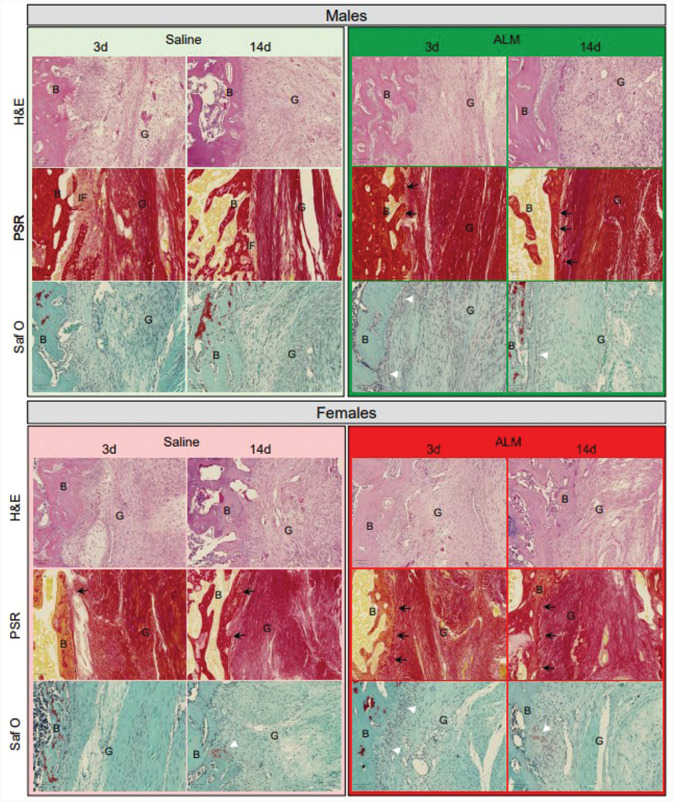
Histological changes associated with anterior cruciate ligament (ACL) graft healing in male and female adenosine, lidocaine, and Mg^2+^ (ALM)–treated and saline control animals, 28 days after early (3 days) and delayed (14 days) ACL reconstruction surgery. Representative images of hematoxylin and eosin (H&E)–, picrosirius red (PSR)–, and safranin O/fast green (SafO)–stained sections of ACL graft within bone tunnels. Black arrows indicate Sharpey fibers, and white arrowheads indicate fibrocartilage formation at the bone (B)-graft (G) interface. Scale bars: 100 μm.

**Figure 6.**
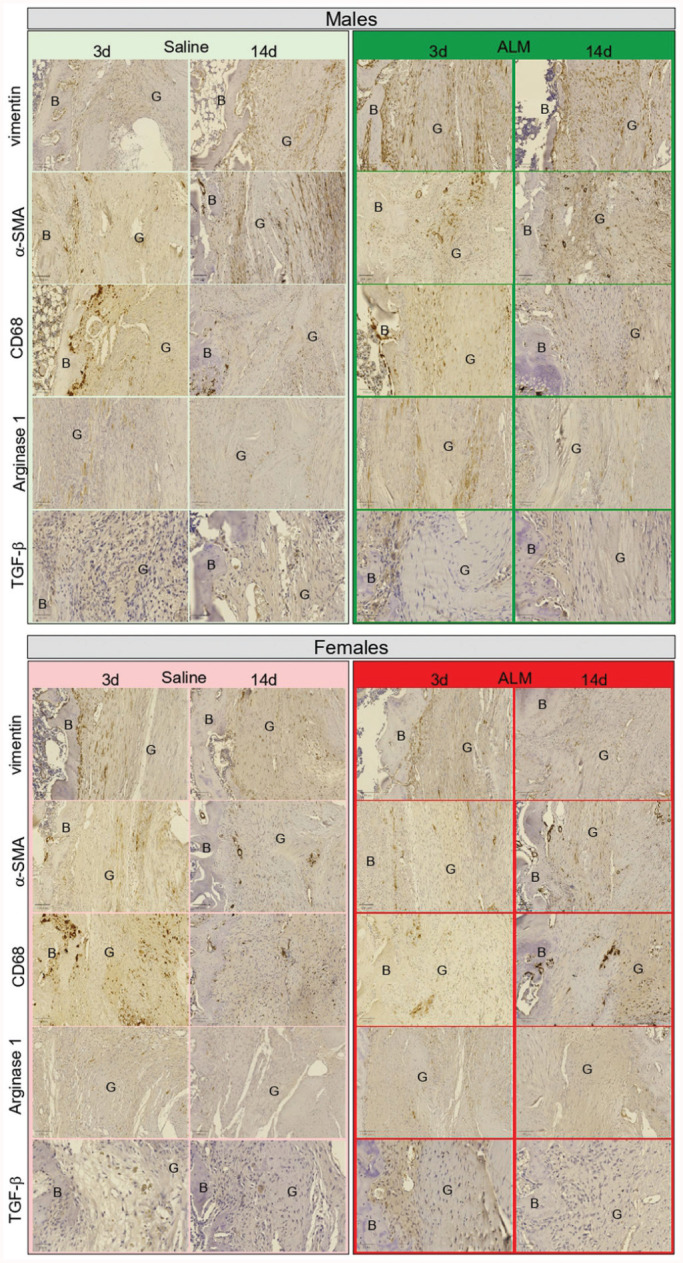
Immunohistochemical staining of anterior cruciate ligament (ACL) grafts of male and female adenosine, lidocaine, and Mg^2+^ (ALM)–treated and saline control animals, 28 days after early (3 days) and delayed (14 days) ACL reconstruction surgery. Representative images of vimentin-, alpha-smooth muscle actin (α-SMA) CD68-, arginase 1–, and transforming growth factor beta 1 (TGF-β1)–stained sections of ACL graft (G) within bone (B) tunnels. Scale bars: 100 μm.

Next, we compared ACL graft healing 28 days after early and delayed ACLR by measuring the gene expression of key markers of inflammation and tissue repair and assessing graft histopathology. Compared with early ACLR, healing appeared more advanced in male ACL grafts after delayed surgery, as evidenced by the increased expression of ECM synthesis markers (Tgfb1, 3.4-fold, *P* > .05; Col3a1, 3.5-fold, *P* = .01; Eln, 2.3-fold, *P* > .05; Fn1, 2.1-fold, *P* = .04) (Appendix Figure A5, available online) and increased presence of Sharpey fibers and narrowing of the fibrovascular interface within bone tunnels (Table 1, Figure 5). Compared with saline controls, markers of ECM synthesis (Tgfb1, 6.8-fold, *P* = .002; Fgf1, 17.3-fold, *P* = .03; Timp1, 2.2-fold, *P* > .05) were decreased in grafts from ALM-treated males to levels comparable to those in native ACL tissue after delayed surgery (Figure A5). Histologically, this corresponded to further narrowing of the fibrovascular interface, increased mucoid degeneration and presence of cells with spindle-shaped nuclei within graft tissue, and more abundant Sharpey fibers, fibrocartilage and bony ingrowth within the bone tunnels of ALM-treated males (Table 1, Figure 5). Compared with saline controls, the percentage of cells staining positive for vimentin (52% vs 33%; *P* > .05), TGF-β (4% vs 0.9%; *P* > .05), and arginase I (5% vs 1%; *P* > .05) also tended to be higher, and α-SMA (4% vs 10%; *P* > .05) lower, within graft tissue from ALM-treated males after delayed surgery (Figure 6; Appendix Figure A6, available online).

**Table 1:** ACL Graft Histopathology Scores for Male and Female ALM-Treated and Saline Control Animals 28 Days After Early (3-Day Delay) or Delayed (14-Day Delay) ACL Reconstruction Surgery*^a^*

	Male				*P* Value	Female				*P* Value
	3-Day Delay		14-Day Delay			3-Day Delay		14-Day Delay		
	Saline (n = 5)	ALM (n = 6)	Saline (n = 8)	ALM (n = 9)		Saline (n = 5)	ALM (n = 6)	Saline (n = 9)	ALM (n = 9)	
ACL graft, central	9.3 (7.6, 9.5)	8.4 (8.1, 9.5)	9.1 (8.3, 10.1)	9.8 (8.3, 10.6)	.40	8.8 (7.9, 10)	9.1 (8.3, 9.3)	8.8 (8.3, 9.9)	9.3 (8.1, 9.5)	.97
Cellularity	2 (1.5, 2.3)	2.3 (1.9, 2.5)*^b^*	2.3 (2, 2.5)	2 (1.8, 2.5)	.75	2 (1.5, 2.3)	2 (1.5, 2.5)	2 (1.5, 2.3)	2 (1.5, 2.5)	.92
Cell morphology	3 (2.5, 3)	2 (1.8, 2)*^b^*	2 (2, 2.8)	2 (2, 3)	**.05**	2 (2, 2.5)	2 (1.8, 2.3)	2 (2, 2)	2 (2, 2)	.66
Collagen fiber orientation	2 (1.8 (2)	1.8 (1.5, 1.8)	2 (1.8, 2)	1.8 (1.5, 2)	.19	1.8 (1.5, 1.9)	1.5 (1.5, 1.8)	1.8 (1.6, 2)	1.8 (1.5, 2)	.27
Mucoid degeneration	0.5 (0.5, 0.8)	1 (0.9, 1.6)	1 (1, 1.5)	1.5 (1, 2)	**.01**	1.5 (1, 1.5)	1.5 (1, 1.6)	1.5 (1, 2)	1.5 (1, 1.8)	.85
Angiogenesis	1.5 (1.3, 1.8)	1.8 (1.4, 2.1)	1.5 (1.5, 2)	2 (1.8, 2)	.2	1.5 (1.5, 2.3)	2 (1.5, 2)	2 (1.5, 2)	2 (1.5, 2.3)	.99
Femur, graft-to-bone	7.5 (7, 8)	6.5 (5.5, 7.1)	6.5 (6, 7)	5.5 (5, 6)	**.002**	6 (5.8, 7.3)	5 (4.5, 6.1)	5.5 (5, 6)	5 (4.3, 5.8)	**.05**
Interface transition	2 (2, 2.3)	1.5 (1.5, 2)	2 (1.6, 2)	1.5 (1.5, 1.8)	**.01**	2 (1.5, 2.3)	1.5 (1.4, 1.6)	1.5 (1.3, 1.5)	1 (1, 1.8)	.11
Interface cellularity	2 (1.5, 2)	1.5 (1, 1.75)	1.5 (1.1, 2)	1.5 (1, 1.8)	.32	1 (1, 1.5)	1 (0.9, 1.1)	1.5 (1.3, 2)	1.5 (1, 1.5)	**.04**
Bony ingrowth	2.5 (2.3, 2.5)	2 (2, 2.1)	2 (2, 2)	1.5 (1.5, 1.8)	**<.001**	2 (2, 2.3)	1.5 (1.5, 2)	1.5 (1.5, 1.5)*^c^*	1.5 (1, 1.5)	**.001**
Giant cells	1 (1, 1.5)	1 (1, 1.5)	1 (1, 1.4)	1 (1, 1)	.23	1 (1, 1.5)	1 (1, 1.1)	1 (1, 1)	1 (1, 1)	.08
Tibia, graft-to-bone	7 (7, 8.3)	6.3 (5.9, 7)	6.5 (6, 7)	6 (6, 7)	**.05**	6 (5.5, 6.8)	5.3 (5, 5.8)	6 (5.8, 6.5)	5 (4.3, 6)	**.03**
Interface transition	2.5 (2.3, 2.5)	1.8 (1.5, 2)*^b^*	2 (1.6, 2)	2 (2, 2.5)	**.007**	2 (1.5, 2)	1.5 (1.5, 1.6)	2 (1.8, 2)	1.5 (1.3, 1.8)*^d^*	**.03**
Interface cellularity	1.5 (1, 1.8)	1 (1, 1.8)	2 (1.5, 2)	1.5 (1, 1.5)	.15	1 (0.8, 1.5)	1 (1, 1.1)	1.5 (1, 1.8)	1 (1, 1)	.07
Bony ingrowth	2.5 (2.5, 2.5)	2 (2, 2.5)	2 (1.5, 2)*^c^*	1.5 (1, 2)	**.002**	2 (2, 2.5)	1.8 (1.5, 2)	1.5 (1.5, 2)	1.5 (1, 1.8)	**.02**
Giant cells	1 (1, 1.5)	1 (0.9, 1.5)	1 (1, 1)	1 (1, 1.5)	.63	1 (1, 1)	1 (1, 1)	1 (1, 1)	1 (1, 1.3)	.2

a Data are presented as median (IQR). The Kruskal-Wallis test and Dunn post hoc analysis were used. Bold *P* values indicate statistical significance. ACL, anterior cruciate ligament; ALM, adenosine, lidocaine, and Mg^2+^.

b *P* < .05, 3-day ALM compared with 3-day saline.

c *P* < .05, 3-day saline compared with 14-day saline.

d *P* < .05, 14-day ALM compared with 14-day saline.

In females, decreases in Tgfb1 (3.8-fold; *P* > .05) and Col1a1 (5.6-fold; *P* = .03) and increases in Fgf1 (2.9-fold; *P* > .05) and Eln (2-fold; *P* > .05) expression were shown in graft tissue and corresponded to more abundant Sharpey fibers and fibrocartilage within bone tunnels after delayed ACLR, compared with early surgery (Table 1, Figure 5). After delayed surgery, the expression of growth factors associated with ECM synthesis (Tgfb1, 8.7-fold, *P* > .05; Fgf1, 7-fold, *P* > .05) tended to be lower in ALM-treated than saline control females (Appendix Figure A5, available online). Histologically, this corresponded with further increases in the presence of Sharpey fibers, tissue continuity, and fibrocartilage formation at the bone-graft interface (Table 1, Figure 5). In females, the percentage of vimentin-positive cells was lower in graft tissue after delayed compared with early ACLR surgery (25% vs 44%; *P* > .05), and reduced further in ALM-treated animals (7% vs 25%; *P* > .05). In addition, cells positive for TGF-β tended to be lower (6% vs 12%; *P* > .05) and arginase I–positive cells higher (5% vs 1%; *P* > .05) in graft tissue from ALM-treated than saline control females after delayed surgery (Figure 6; Appendix Figure A6, available online). Together, these data suggest that ACL graft maturation is more advanced in females than males and is improved further by delayed surgery and ALM treatment.

### Articular Cartilage Degeneration

**Figure 7.**
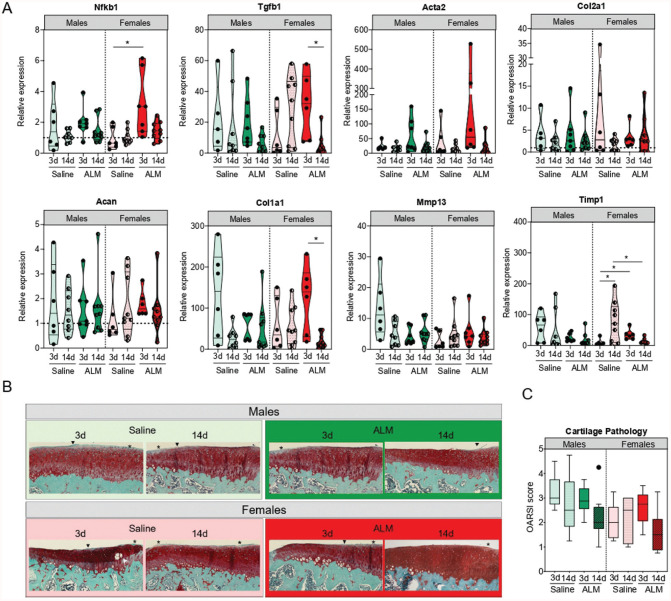
Articular cartilage remodeling in male and female adenosine, lidocaine, and Mg^2+^ (ALM)–treated and saline control animals, 28 days after early (3 days) and delayed (14 days) anterior cruciate ligament reconstruction surgery. (A) Relative expression of markers of inflammation (nuclear factor kappa B [Nfkb]), chondrocyte activation and proliferation (transforming growth factor beta 1 [Tgfb1] and actin alpha 2 [Acta2]), extracellular matrix (ECM) components (collagen type 2 alpha 1 chain [Col2a1] and aggrecan [Acan]), fibrocartilage (collagen type 1 alpha 1 chain [Col1a1]), and ECM remodeling enzymes (matrix metalloproteinase 13 [Mmp13] and tissue inhibitor of MMP-1 [Timp1]) in medial articular cartilage. (B) Representative safranin O/fast green–stained sections. (C) Osteoarthritis Research Society International (OARSI) scoring (of a total possible score of 15) of medial articular surfaces. Minor proteoglycan loss (asterisk in panel B) and surface fibrillation (arrowhead) were evident with no sex, surgical timing, or treatment-specific differences. Data are shown as median and IQR. *P < .05. Kruskal-Wallis test, Dunn post hoc analysis.

Finally, given the increased risk of PTOA progression after ACLR,^5,15,61^ we compared early molecular markers of cartilage remodeling and the histopathology of articular cartilage 28 days after early and delayed ACLR. No significant sex, timing, or treatment differences were observed in expression levels of the major ECM constituents or articular cartilage, Col2a1 and Acan. In males, there were no statistically significant timing or treatment differences in cartilage degeneration markers at 28 days postoperatively. In females, the expression of Timp1, an inhibitor of catabolic Mmp13 activity, was 9.8-fold higher (*P* = .01) in articular cartilage after delayed ACLR compared with early ACLR. Compared with saline controls, the expression of Timp1 (7.1-fold; *P* = .048) and Tgfb1 (20.4-fold; *P* > .05), markers of chondrocyte proliferation, was lower in ALM-treated females after delayed surgery (Appendix Figure 7A, available online). Histologically, mild degenerative changes were apparent in the articular cartilage of both sexes, including focal areas of decreased matrix staining in the superficial layer, and occasional surface fibrillation (Figure 7B). While minor improvements were shown in OARSI scores for ALM-treated males and females after delayed surgery, these changes were not statistically significant (Figure 7C).

## Discussion

Our findings support the hypotheses that surgery-induced immunoinflammatory reactivity is reduced when ACLR is delayed from 3 to 14 days, and that perioperative ALM therapy modulates early immunoinflammatory processes, regardless of sex and timing. In a clinically relevant rat model of ACL rupture and reconstruction, we report the following: (1) delaying surgery from 3 to 14 days led to less systemic inflammation and improved joint tissue repair after surgery; (2) irrespective of surgical timing, healing in the ACL graft and adjacent joint tissue appeared to be more advanced in females than males based on joint histopathology and molecular profiles; (3) perioperative ALM therapy blunted surgery-induced immunoinflammatory reactivity in the early postoperative period; and (4) ALM also appears to augment intrinsic pro-healing responses within the ACL graft of males and females, regardless of surgical timing. We now discuss these major findings.

### Sex Differences in the Systemic Inflammatory Response to ACL Rupture

An interesting finding was that the acute systemic inflammatory response to ACL rupture was sex-specific, with females exhibiting faster resolution of this response than males. For example, at 2 days after injury, while circulating neutrophils and plasma inflammatory mediators (IL-6, TNF-α, IL-1β, and MCP-1) had returned to baseline levels in females, persistent neutrophil and monocyte recruitment was evident in males. In addition, by day 13 after injury (no surgery) females had lower numbers of circulating T cells, monocytes, lymphocyte chemoattractant, and IP-10 compared with baseline levels, suggesting a faster resolution of the inflammatory response in the ACL-ruptured knee. This is consistent with a previous study showing that females appeared to have a more advanced stage of joint tissue repair than males at 31 days after injury in a rat model of noninvasive ACL rupture.^39^ Together, our data show sex differences in the timing and cellular composition of the systemic immunoinflammatory response to ACLR surgery, and are consistent with recent studies suggesting accelerated resolution of acute inflammation in females compared with males.^35,50,54^

### Delayed Surgery Led to Reduced Pre- and Postoperative Systemic Inflammation

Another important finding of the present study was that delayed surgery (14 days after ACL injury) had the advantage of diminishing the systemic immunoinflammatory response in both sexes, compared with ACLR surgery after 3 days. Delayed surgery was associated with reduced inflammatory cell recruitment, evidenced by lower plasma IL-1β and MCP-1 levels, and circulating neutrophil and classic monocyte frequencies. Furthermore, classic monocytes, T_Helper_ and T_Regulatory_ cell numbers remained significantly lower than baseline levels in peripheral blood of males and females to day 5 postoperatively, indicative of a mononuclear cell— rather than inflammatory cell—dominant immune microenvironment within the operated knee, which is consistent with the contrasting systemic profile shown preoperatively (day 2 vs day 13 post-ACL rupture).

Our study further showed that delaying surgery led to improved clinical outcomes in both sexes. Compared with early ACLR, recovery of preoperative body weight was faster, joint pain and swelling reduced sooner, and walking gait mechanics improved after delayed surgery in both males and females. Differences in recovery trajectories were reflected systemically in hematology and immune mediator profiles at day 28 postoperatively. In contrast to early ACLR,^42^ there was no evidence of persistent systemic inflammation in males or females, 28 days after delayed surgery. In both sexes, an advanced healing phenotype was associated with higher plasma levels of anti-inflammatory and pro-healing cytokines (IL-6, IL-1β, and IL-4) 28 days after delayed surgery.

### ALM Therapy Promotes Earlier Resolution of Systemic Inflammation After ACLR Surgery

A novel finding of this study was that regardless of ACLR timing, perioperative ALM therapy appears to dampen the surgery-induced systemic inflammatory response in a sex-specific manner. In males, ALM reduced circulating neutrophils and NK cell numbers and plasma concentrations of the monocyte chemoattractant, MCP-1, and increased plasma IL-4 and IL-18 levels after delayed ACLR, suggesting a shift from a pro-inflammatory to an anti-inflammatory and immunoregulatory state.^26^ In females, ALM had a differential effect in boosting circulating NK cell numbers, which possess immunoregulatory properties, consistent with earlier resolution of the acute inflammatory response to surgery already discussed.^28^ Interestingly, after early ACLR, ALM exerts a different immunomodulatory effect compared with delayed ACLR. In females, ALM blunted neutrophil activation and plasma IL-6 and TNF-α levels, whereas ALM reduced monocyte recruitment and plasma IL-1β responses in males.^44^ This differential ALM sex-specific effect may be due to (1) distinct joint tissue healing phenotypes of males and females at 2 and 13 days after ACL rupture, and (2) sex-specific responses to the trauma of ACLR surgery. In summary, the effect of perioperative ALM therapy to resolve inflammation appears to be sex-specific and depends on the timing of surgery.

### ALM Dampens Joint Inflammation in Both Sexes and Augments Graft Tissue Repair

Our study further showed that ALM reduced synovial levels of inflammatory mediators (TNF-α and IL-1β) in both sexes at 28 days postoperatively, regardless of surgical timing. Together, molecular and histological analyses supported a more advanced healing phenotype within the ACL graft of ALM-treated males and females, albeit with distinct healing phenotypes after early and delayed ACLR. Specifically, after early ACLR, ALM appears to increase the expression of tissue repair markers (Tgfb1, Ccn2, Col3a1, and Fn1) in the ACL graft that are associated with a proliferative phase of healing, corresponding to histological evidence of increased angiogenesis, collagen deposition, and improved healing at the bone-graft interface in both sexes.^42^ In contrast, the expression of proliferative phase markers was decreased in the graft tissue of ALM-treated males and females compared with saline controls after delayed ACLR, which is consistent with a slowing of ECM deposition during tissue remodeling and the maturation phases of healing.^12^ A more advanced graft healing profile was supported histologically, with increased collagen fiber orientation, improved continuity and abundance of Sharpey fibers, and bony ingrowth evident within graft tunnels of ALM-treated animals after delayed surgery. Interestingly, ALM also appeared to boost levels of arginase I–positive MDSCs and reparative M2 macrophages in ACL grafts of both sexes after delayed surgery, which was supported by the observation of decreased myeloid cells within draining lymph nodes of ALM-treated male and female animals. MDSCs are derived from polymorphonuclear and monocytic precursors and play a key role in tissue repair and restoring tissue homeostasis.^29,51^ Thus, irrespective of surgical timing, ALM therapy appears to promote a healing phenotype in the graft of both sexes after 28 days compared with saline controls.

While ALM appeared to augment graft healing in both sexes after early and delayed ACLR, a notable finding was that joint tissue reparative processes were enhanced in a sex-specific manner. Consistent with the differential systemic immunoinflammatory responses discussed above, graft molecular profiles and histopathology showed that healing was more advanced in females than males at 28 days postoperatively, which is consistent with previous findings in an experimental model of early ACLR.^42,44^ After delayed ACLR, ALM boosted numbers of vimentin-positive cells and ECM deposition at the bone-graft interface in males. Vimentin is a type 3 intermediate cytoskeletal filament that promotes the migration and proliferation of fibroblasts and mesenchymal stem cells and facilitation of collagen deposition during the proliferative phase of healing.^46^ In contrast, in females ALM increased synovial levels of MIP-1α, a chemokine central to tissue remodeling, and decreased vimentin-positive cells and the gene expression of growth factors associated with the proliferative phase of healing in graft tissue (Tgfb and Fgf1). Histologically, this corresponded to improved continuity and bony ingrowth at the bone-graft interface in ALM-treated females, compared with saline controls. Lastly, contrasting myeloid cell profiles within draining lymph nodes of the operated knee suggest that ALM appears to dampen granulocytic MDSC precursor recruitment in males and monocytic MDSC recruitment in females. MDSC involvement in the ACL repair processes, however, remains poorly understood, and potential sex differences in the MDSC subset contribution to ACL graft healing warrant further investigation. In summary, these data demonstrate a more advanced graft healing phenotype in females than males, and suggest that ALM treatment may enhance the intrinsic sex-specific time course of ACL graft healing after both early and delayed ACLR.

### ALM May Exert Sex-Specific Chondroprotective Changes in Articular Cartilage

In the current study, sex-specific changes associated with surgical timing and ALM treatment were also found in articular cartilage. Despite similar histological appearance, transcriptional profiles reflected ongoing repair and remodeling processes in the articular cartilage of both sexes 28 days after early and delayed ACLR. In males, ALM blunted markers of cartilage degeneration (Col1a1 and Mmp13) after early surgery,^42^ but not after delayed surgery. In females, delayed surgery was associated with higher levels of anabolic (Timp1 and Tgfb1) markers, with ALM appearing to reduce the expression of Timp1, Tgfb1, and Col1a1 to levels comparable to those in the uninjured knee. Their collective decrease and corresponding minor improvements in histopathology support the notion that repair processes are more advanced in the articular cartilage of females than males, consistent with the ACL graft. Together, these data highlight differences in articular cartilage healing profiles of males and females in response to early and delayed ACLR and suggest that perioperative ALM therapy exerts sex-specific chondroprotective changes within the knee. Longer-term studies are required to determine whether these early molecular changes provide ongoing protection in both sexes.

### Clinical Relevance

Our study addressed 3 key contributors to improve clinical outcomes after ACLR: the timing of surgery, sex differences in postoperative healing, and the use of perioperative immunomodulatory therapy to provide whole-joint protection. The early and delayed surgery arms used in this rat model were selected to represent real-world timelines for ACLR after injury and diagnosis. Deferred diagnosis of ACL injury is not uncommon, and poor initial treatment can render patients unfit for surgery until range of motion and quadriceps control has been restored and effusion settled. Furthermore, global increases in elective surgery wait times^49^ mean that injury-to-ACLR surgery times can extend to a year or more, particularly within public health care systems. Our study supports the reduced postoperative systemic inflammatory benefits of delaying surgery after ACL rupture with improved joint tissue repair. Importantly, it also highlights sex differences in healing profiles after ACL injury and surgery, which requires further investigation. Sex differences in both the timing and phenotype of immunoinflammatory and tissue repair processes after injury and surgery are underappreciated in the clinical setting. Furthermore, our study demonstrates that irrespective of surgical timing, intraoperative ALM therapy appears to dampen early local and systemic inflammatory responses and may afford joint protection to both sexes after experimental ACLR. Because there are no drug therapies that protect the whole joint with improved patient outcomes, future human trials will examine the safety of ALM during ACLR surgery.

### Limitations

We acknowledge several limitations to the study. First, the use of a rat model necessitates open joint surgery, rather than the more common arthroscopic approach used clinically. Second, joint tissue healing was evaluated at a single time point, with the study follow-up period limited to 28 days postoperatively, by which time complete graft maturation is unlikely to have been achieved.^9^ Third, an endpoint of 28 days may also be too early to provide histological evidence of PTOA.^3,36^ Fourth, we used the contralateral, uninjured, and nonoperated knee as our statistical comparator for cellular and molecular analysis of joint inflammation and tissue repair markers, which others suggest may be unsuitable due to potentially altered limb loading.^55^ Fifth, the biomechanical strength of the graft was not assessed since graft maturity and osseointegration are achieved between 6 and 15 weeks after implantation.^9^ Lastly, given the inherent biological differences between individual animals and responses to experimental procedures, the study may have lacked statistical power to detect subtle differences in tissue repair profiles and joint function between sexes and treatment groups. Despite these limitations, the molecular and histopathological changes in this model are consistent with those described clinically, strengthening its utility to examine sex-specific effects of ACL injury and surgery, and new perioperative drug therapies to improve postoperative outcomes.

## Conclusion

Delayed ACLR surgery was associated with improved recovery and joint healing in both sexes. Regardless of surgical timing, females appear to have a faster rate of healing within the operated joint than males. We further showed that perioperative ALM therapy blunts postoperative immunoinflammatory responses in both sexes, which may augment joint healing and provides support for clinical transition.

## Supplemental Material

sj-pdf-1-ajs-10.1177_03635465251383556 – Supplemental material for Delayed Surgery and Adenosine, Lidocaine, and Mg2+ Immunomodulatory Therapy Improve Joint Recovery in a Sex-Specific Manner After Anterior Cruciate Ligament Reconstruction in a Rat Model Supplemental material, sj-pdf-1-ajs-10.1177_03635465251383556 for Delayed Surgery and Adenosine, Lidocaine, and Mg2+ Immunomodulatory Therapy Improve Joint Recovery in a Sex-Specific Manner After Anterior Cruciate Ligament Reconstruction in a Rat Model by Jodie L. Morris, Hayley L. Letson, Peter C. McEwen and Geoffrey P. Dobson in The American Journal of Sports Medicine

## Footnotes

∥ References 1, 4, 27, 30, 38, 52, 56, 60, 65.
