# Supplementary material for: Delayed Surgery and Adenosine, Lidocaine, and Mg2+ Immunomodulatory Therapy Improve Joint Recovery in a Sex-Specific Manner After Anterior Cruciate Ligament Reconstruction in a Rat Model
Source: Am J Sports Med. 2025 Oct 23;53(13):3166–81. doi: 10.1177/03635465251383556 (PMC12578965; doi:10.1177/03635465251383556)
Supplement: sj-pdf-1-ajs-10.1177_03635465251383556 – Supplemental material for Delayed Surgery and Adenosine, Lidocaine, and Mg2+ Immunomodulatory Therapy Improve Joint Recovery in a Sex-Specific Manner After Anterior Cruciate Ligament Reconstruction in a Rat Model [file sj-pdf-1-ajs-10.1177_03635465251383556.pdf]

Table A1. Number of animals assessed for each metric.

	Metrics	Male				Female			
		3d delay		14d delay		3d delay		14d delay	
		Saline	ALM	Saline	ALM	Saline	ALM	Saline	ALM
Systemic	Body weight recovery	10	11	8	9	10	11	9	9
	Intraoperative parameters	10	11	8	9	10	11	9	9
	Complete blood count	10	11	8	9	10	11	9	9
	Peripheral blood, flow cytometry	10	11	8	9	10	11	9	9
	Plasma cytokines and chemokines	8	8	8	9	8	8	8	9
Joint	Swelling	10	11	8	9	10	11	9	9
	Gait assessment	10	11	8	9	10	11	9	9
	Sensory testing	10	11	8	9	10	11	9	9
	Knee extension angle	10	11	8	9	10	11	9	9
	Quadriceps atrophy	10	11	8	9	10	11	9	9
	Macroscopic pathology	10	11	8	9	10	11	9	9
	Synovial cytokines and chemokines	6	7	8	9	6	7	9	9
	Histopathology	5	6	8	9	5	6	9	9
	Gene expression profiling	6	7	8	9	6	7	9	9

Table A2. Flow cytometry antibodies for leukocyte phenotyping.

Antigen	Clone	Fluorochrome	Dilution	Manufacturer
CD45	OX-1	APC-Cy7	1 in 200	Becton Dickinson
CD45RA	OX-33	BV480	1 in 140	Becton Dickinson
CD3	IF4	APC	1 in 200	Becton Dickinson
CD4	OX-35	PE-Cy7	1 in 200	Becton Dickinson
CD8a	OX-8	BB700	1 in 170	Becton Dickinson
CD161a	10/78	FITC	1 in 140	Becton Dickinson
CD25	OX-39	BV421	1 in 140	Becton Dickinson
Foxp3	150D	PE	1 in 25	Biolegend
CD43	W3/13	PE-Cy7	1 in 140	Biolegend
CD62L	HRL1	PE	1 in 150	Becton Dickinson
CD11b/c	OX-42	BV421	1 in 200	Becton Dickinson
His48	His48	FITC	1 in 150	Becton Dickinson
RT1B	OX-6	PE	1 in 140	Becton Dickinson

Table A3. Histopathology grading scheme for assessment of ACL graft healing within the central region, and femoral and tibial bone tunnels, with each parameter scored using a scale of 0 (normal architecture) to 3 (severe pathology).

**Parameter**		**Feature**
**ACL, central region**		
1	Cellularity	Describing the infiltration of inflammatory and non-inflammatory cells
2	Cell morphology	Describing the relative abundance of cells with round- versus spindle-shaped nuclei
3	Collagen fiber orientation	Indicating the degree of longitudinal alignment of collagen bundles
4	Mucoid degeneration	Describing the presence of necrosis, separation and disorganization of collagen fibers
5	Angiogenesis	Indicating the degree of graft revascularization
**ACL, bone tunnels**		
1	Interface transition	Indicating the degree of bone-graft continuity, abundance of Sharpey's fibres and fibrocartilage
2	Interface cellularity	Describing the degree of cell recruitment to the bone-graft interface
3	Bony ingrowth	Indicating the formation of new bone around the tunnel
4	Giant cells	Describing the abundance of multinucleated cells at the interface

Table A4. Primers used for gene expression analysis.

Gene	QuantiTect Primer Assay (Qiagen)
Acan	QT00189518
Acta2	QT01615901
Ccn2	QT00182021
Col1a1	QT00366016
Col2a1	QT01084118
Col3a1	QT00365981
Eln	QT00413007
Fgf1	QT00194551
Fn1	QT00179333
Hprt1	QT00199640
Mmp9	QT00178290
Mmp13	QT01629593
Nfkb1	QT00370545
Tgfb1	QT00187796
Timp1	QT00185304

Acan, aggrecan; *Acta2,* actin alpha 2, smooth muscle; *Ccn2,* cellular communication network factor 2; *Col1a1,* collagen type I alpha 1 chain; *Col2a1,* collagen type II alpha 1 chain; *Col3a1,* collagen type III alpha 1 chain; *Eln,* elastin; *Fgf1*, fibroblast growth factor 1; *Fn1,* fibronectin 1; *Hprt1,* hypoxanthine phosphoribosyltransferase 1 (housekeeping gene); *Mmp9,* matrix metalloproteinase 9; *Mmp13,* matrix metalloproteinase 13; *Nfkb1,* nuclear factor kappa B subunit 1; *Tgfb1,* transforming growth factor, beta 1; *Timp1,* TIMP metallopeptidase inhibitor 1.

Table A5. Anterior cruciate ligament (ACL) injury profiles and operative metrics for ALM-treated and Saline control male and female animals following 3-day or 14-day delay between ACL rupture and reconstruction surgery.

	**Male**					**Female**				
	**3d delay**		**14d delay**		***p* value**	**3d delay**		**14d delay**		***p* value**
	**Saline**	**ALM**	**Saline**	**ALM**		**Saline**	**ALM**	**Saline**	**ALM**	
**ACL rupture profile (n)**										
complete (partial)	9 (1)	9 (2)	7 (1)	9 (0)	0.765	9 (1)	9 (2)	7 (2)	6 (3)	0.677
proximal (mid-substance)	10 (0)	10 (1)	8 (0)	8 (1)	0.844	9 (1)	11 (0)	8 (1)	8 (1)	0.686
**Surgery time (min)**[1^]	47.8 (7.3)	49.3 (8.1)	53.9 (4.5)	51.7 (6.1)	0.364	49.4 (7.1)	47.1 (4.9)	49.2 (5.8)	52.3 (12.2)	0.229
**Blood loss (g)**[1#]	1.25 (0.62)	1.1 (0.34)	1.04 (0.86)	0.76 (0.42)	0.183	0.64 (0.25)	0.69 (0.33)	0.53 (0.28)	0.61 (0.31)	0.490

[1]Values represent mean (standard deviation).
^Inclusive of catheter insertion, tendon harvest, ACLR surgery and catheter removal.
#Approximates 3.8% and 3.9% of total blood volume for male and female rats, respectively.
Kruskal-Wallis with Dunn's post-hoc test.

Table A6. Comparison of hind limb ventral temporal-spatial gait parameters for ALM-treated and Saline control male and female animals 28-days after early and delayed ACLR surgery.

Indices	Sex	Treatment Group	ACLR Timing 3d delay	ACLR Timing 14d delay
Stance width, cm	Male	Saline	5.8 (0.2)	5.2 (0.3)^
		ALM	6.1 (0.2)	5.2 (0.1)^
	Female	Saline	4.5 (0.1) †	4.5 (0.2)
		ALM	4.8 (0.1)	4.5 (0.2)
Stride length[a], cm	Male	Saline	17.6 (0.7)	14.8 (0.7)^
		ALM	17.8 (0.7)	15.8 (0.5)^
	Female	Saline	16.5 (0.7)	14.7 (0.4)
		ALM	18.9 (0.6)*	16.3 (0.8)^
Stride length variability[a], %	Male	Saline	13.5 (1.6)	14.0 (1.7)
		ALM	12.6 (1.1)	13.6 (2.0)
	Female	Saline	12.7 (0.5)	11.0 (1.9)
		ALM	10.1 (1.3)	13.4 (2.7)
Stride time[a], ms	Male	Saline	497 (41)	565 (62)
		ALM	464 (28)	537 (49)
	Female	Saline	402 (35)	441 (21)
		ALM	349 (31)	413 (35)
Stride time variability[a], %	Male	Saline	25.8 (5.3)	29.0 (3.1)
		ALM	27.3 (2.7)	29.3 (4.1)
	Female	Saline	21.1 (3.5)	23.3 (1.6)
		ALM	18.8 (3.6)	20.9 (3.0)
Step length, cm	Male	Saline	10.5 (0.2)	9.1 (0.2)^
		ALM	10.6 (0.3)	9.4 (0.2)^
	Female	Saline	9.4 (0.3) †	8.5 (0.2)^
		ALM	10.5 (0.3)*	9.3 (0.4)^
Paw angle[a], degrees	Male	Saline	0.5 (0.1)	0.7 (0.2)
		ALM	0.5 (0.1)	0.5 (0.1)
	Female	Saline	0.8 (0.3)	0.8 (0.1)
		ALM	0.5 (0.1)	0.7 (0.1)
Track Speed, cm/s	Male	Saline	38.1 (3.9)	29.2 (3.8)
		ALM	39.8 (3.7)	31.6 (2.7)
	Female	Saline	44.6 (5.0)	33.7 (1.9)
		ALM	58.2 (5.2)#	41.9 (4.5)^

Data show mean (SEM).
Two-way ANOVA, Tukey's test.
[a]Operated (right) hind limb values.
*$p < 0.05$ ALM compared to Saline.
^$p < 0.05$ Early compared to Delayed ACLR.
#$p < 0.05$ ALM male compared to ALM female.
†$p < 0.05$ Saline male compared to Saline female.

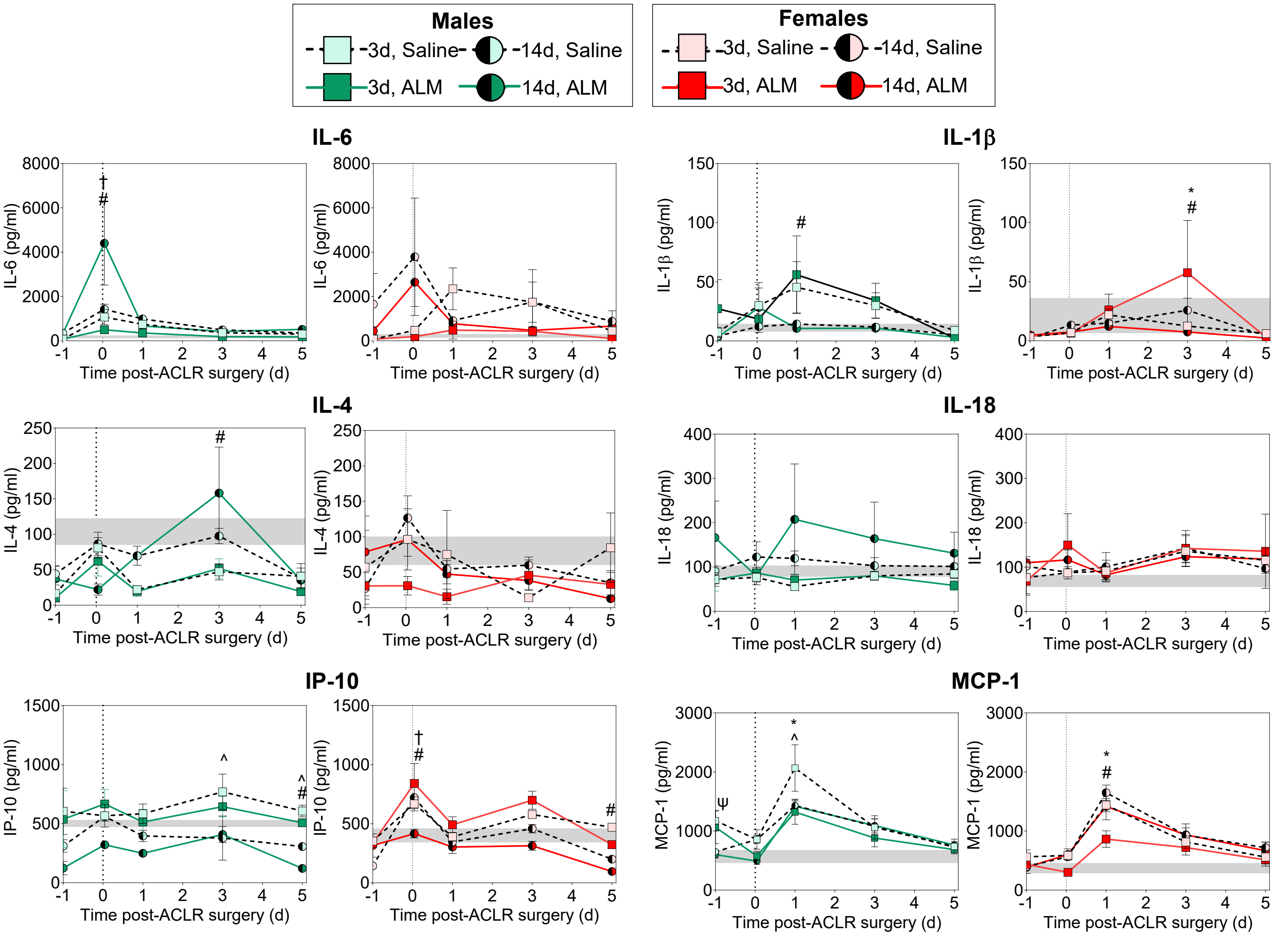

Figure A1. Systemic inflammatory cytokine and chemokine responses in male and female ALM-treated and Saline control animals after early (3-days) and delayed (14-days) ACLR surgery. Data show mean ± SEM. n=8-11 per sex, per time point * $p < 0.05$, 3d ALM, compared to 3d Saline. ^ $p < 0.05$, 3d Saline, compared to 14d Saline. # $p < 0.05$, 3d ALM, compared to 14d ALM. † $p < 0.05$, 14d Saline, compared to 14d ALM. Ψ $p < 0.05$, 3d Saline, compared to baseline levels. Two-way ANOVA, Tukey post-hoc test. Grey shaded areas in graphs show mean ± SEM for healthy baseline animals (n=8 per sex). Dotted vertical line represents commencement of ACLR surgery (time 0). Interleukin, IL; interferon-gamma induced protein 10, IP-10; monocyte chemoattractant protein 1, MCP-1.

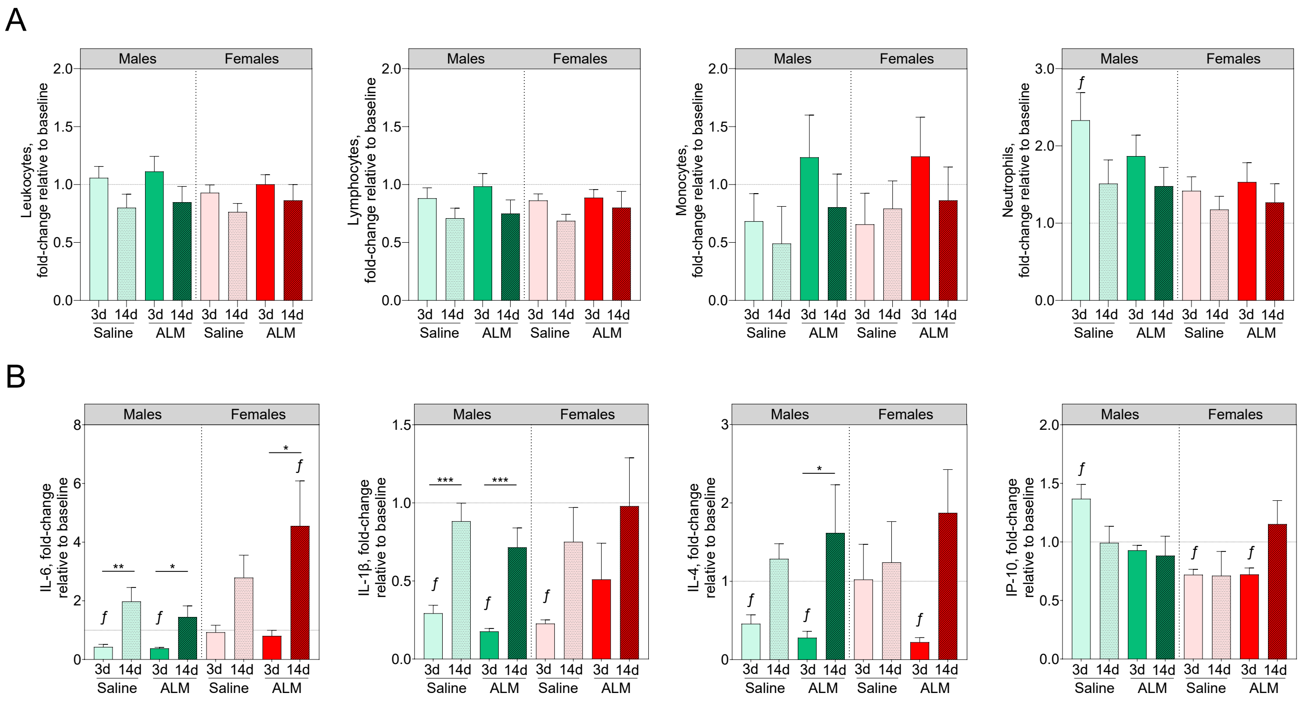

Figure A2. A) Peripheral blood leukocyte subset frequencies and B) plasma inflammatory cytokine and chemokine concentrations in male and female ALM-treated and Saline control animals 28-days after early (3-days) and delayed (14-days) ACLR surgery. Data show mean fold-change relative to baseline levels (indicated by dotted line) ± SEM. n=8-11 per sex, per time point. * $p < 0.05$. ** $p < 0.01$. *** $p < 0.001$. *ƒ* $p < 0.05$, compared to baseline levels. Two-way ANOVA, Tukey post-hoc test. Interleukin, IL; interferon-gamma induced protein 10, IP-10.

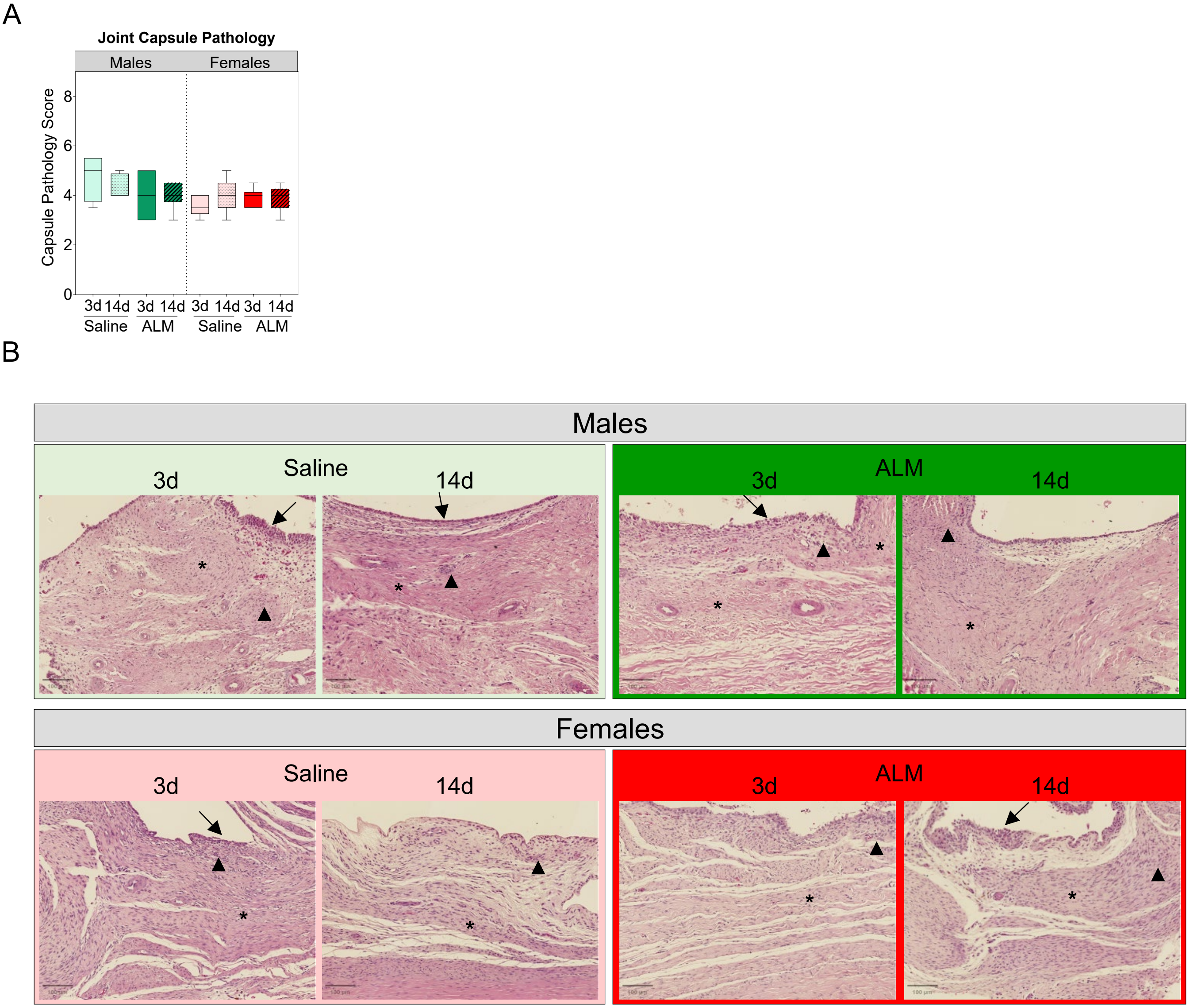

Figure A3. Medial joint capsule histopathology scores of synovitis, inflammatory cell infiltration and fibrosis (out of a total possible score of 9), and B) representative hematoxylin and eosin (H&E)-stained sections of operated knees of male and female ALM-treated and Saline control animals, 28-days after early (3-days) and delayed (14-days) ACLR surgery. Mild synovitis (arrow), cell infiltration (arrowhead), and extracellular matrix deposition (asterisk) was evident in males and females, with no apparent surgical timing-, or treatment-specific differences. Data show median and interquartile ranges. Scale bars: 100 µm.

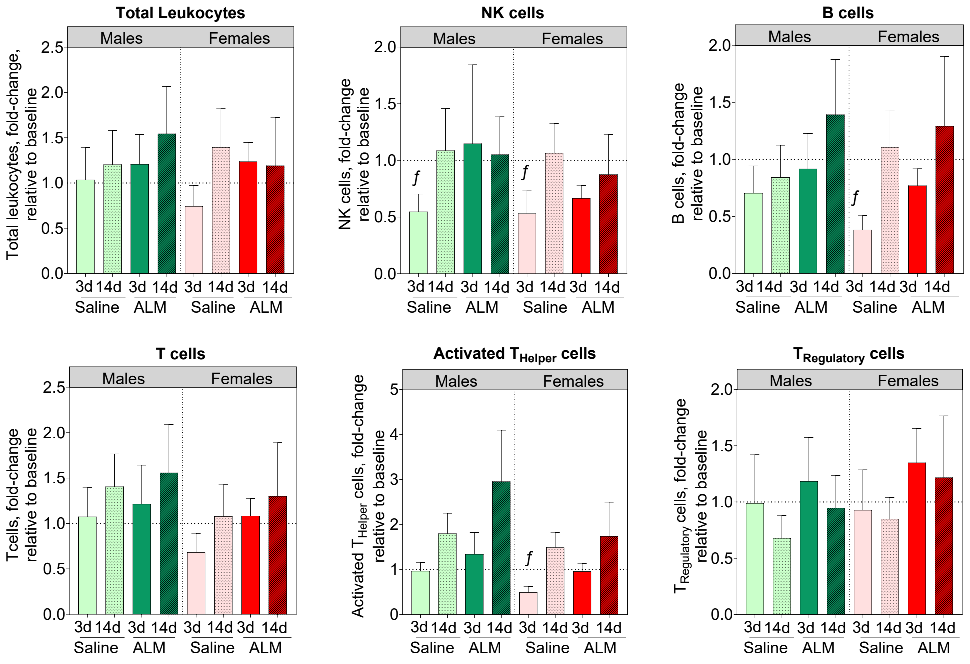

Figure A4. Leukocyte subset frequencies in draining lymph nodes of male and female ALM-treated and Saline control animals, 28-days after early (3-days) and delayed (14-days) ACLR surgery. Data show fold-change relative to draining lymph nodes of non-operated knees (baseline, indicated by dotted line) ± SEM. n=8-11 per sex, per time point. *f* $p < 0.05$, compared to baseline. Two-way ANOVA, Tukey post-hoc test.

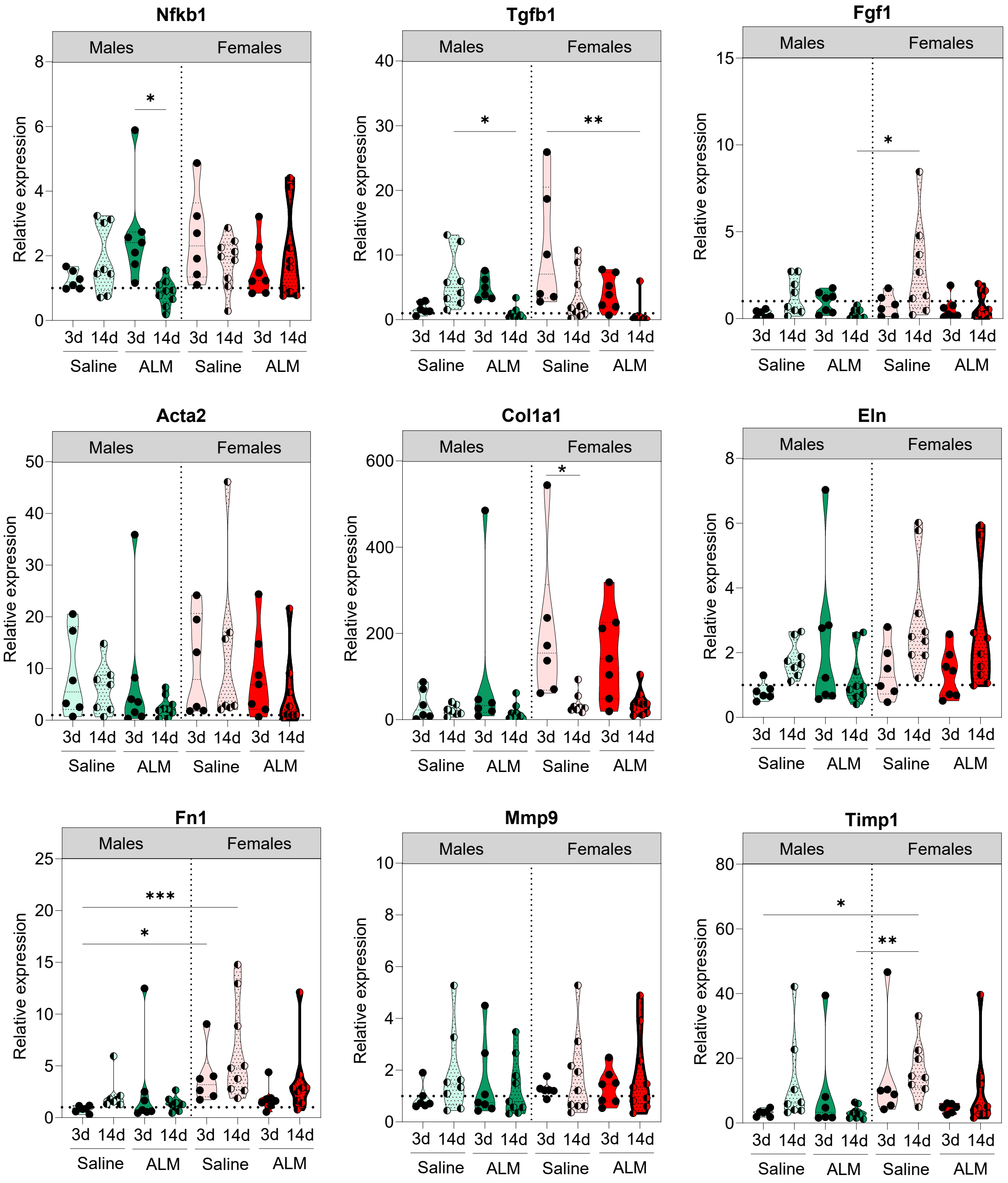

Figure A5. Relative expression of markers of inflammation (nuclear factor kappa B (Nfkb)), stem cell/fibroblast activation (transforming growth factor beta 1 (Tgfb1), fibroblast growth factor 1 (Fgf1), α-smooth muscle actin (Acta2)), extracellular matrix (ECM) components (collagen type I (Col1a1), elastin (Eln), fibronectin (Fn1)), and ECM remodelling enzymes (matrix metalloproteinase-9 (Mmp9), tissue inhibitor of MMP-1 (Timp1)) in ACL graft tissue from male and female ALM-treated and Saline control animals, 28-days after early (3-days) and delayed (14-days) ACLR surgery. Data show median and interquartile ranges. * $p < 0.05$. ** $p < 0.01$. *** $p < 0.001$. Kruskal-Wallis test, Dunn's post-hoc analysis.

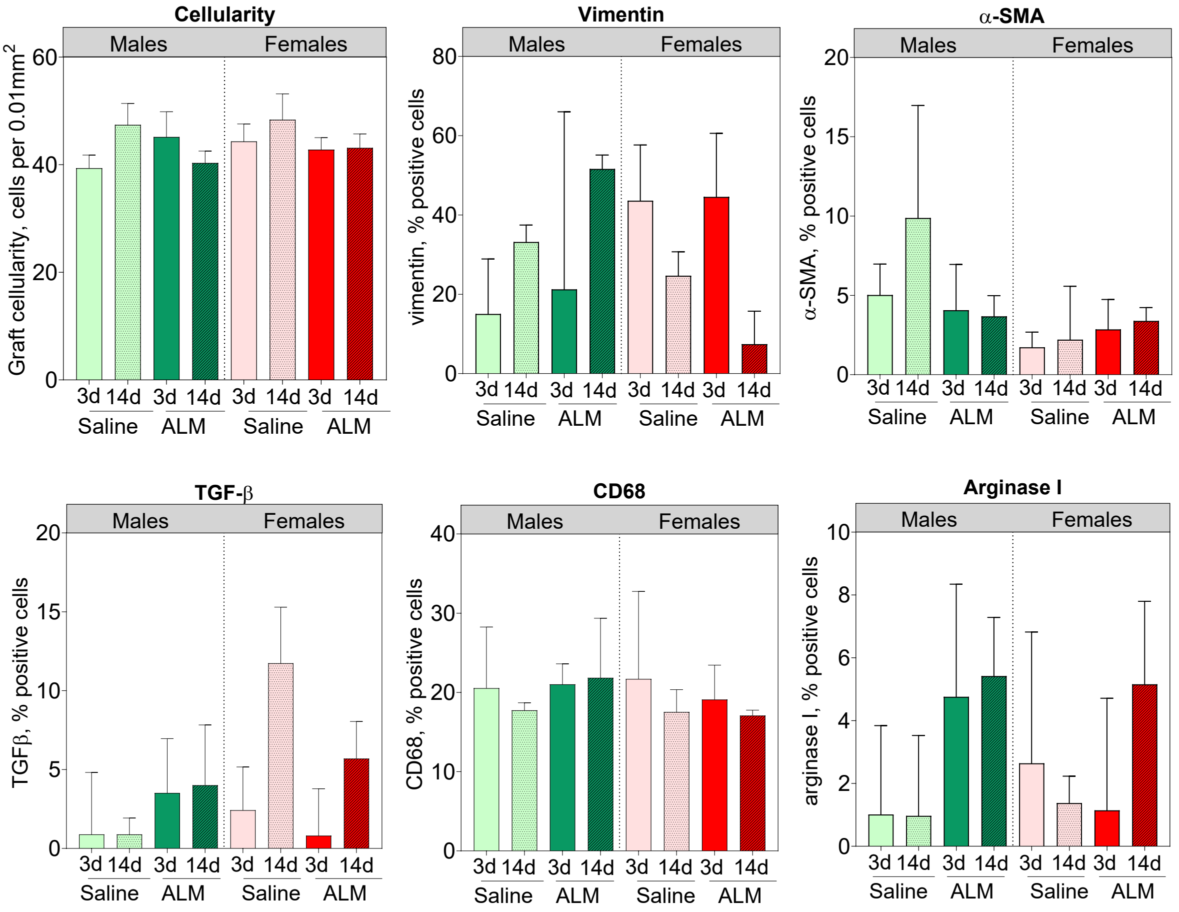

Figure A6. Total cellularity, and the percentage of cells staining positive for vimentin, alpha-smooth muscle actin (α-SMA), transforming growth factor beta (TGF-β1), CD68, and arginase I within ACL graft tissue from male and female ALM-treated and Saline control animals, 28-days after early (3-days) and delayed (14-days) ACLR surgery. Data show median and interquartile range (IQR). Kruskal-Wallis test, Dunn's post-hoc analysis.
